# Impact of symptom duration on the short- and long-term efficacy of bimekizumab in axial spondyloarthritis: results up to 2 years

**DOI:** 10.1186/s13075-026-03729-6

**Published:** 2026-01-19

**Authors:** Sofia Ramiro, Fabian Proft, Raj Sengupta, Astrid van Tubergen, Anna Moltó, Lianne S. Gensler, Mitsumasa Kishimoto, Vanessa Taieb, Sarah Kavanagh, Shawna Evans, Victoria Navarro-Compán

**Affiliations:** 1 Department of Rheumatology, Leiden University Medical Center, Leiden, The Netherlands; 2 Zuyderland Medical Center, Heerlen, The Netherlands; 3 Department of Gastroenterology, Infectiology and Rheumatology (including Nutrition Medicine), Charité – Universitätsmedizin Berlin, corporate member of Freie Universität Berlin and Humboldt-Universität zu Berlin, Berlin, Germany; 4 The Royal National Hospital for Rheumatic Diseases, Bath, UK; 5 Department of Medicine, Division of Rheumatology, Maastricht University Medical Center, Maastricht, The Netherlands; 6 Université Paris Cité, Paris, France; 7 Hôpital Bichat GHU AP-HP.Nord, Paris, France; 8 INSERM U-1153 Center for Research in Epidemiology and Statistics, Paris, France; 9 Department of Medicine/Rheumatology, University of California, San Francisco, San Francisco, CA USA; 10 Department of Nephrology and Rheumatology, Kyorin University School of Medicine, Tokyo, Japan; 11 UCB, Colombes, France; 12 UCB, Morrisville, NC USA; 13 Department of Rheumatology, La Paz University Hospital, IdiPaz, Madrid, Spain

**Keywords:** Axial spondyloarthritis, Radiographic axial spondyloarthritis, IL-17 inhibitors, Bimekizumab, Symptom duration

## Abstract

**Background:**

Bimekizumab, a monoclonal IgG1 antibody that selectively inhibits IL-17F in addition to IL-17A, showed efficacy to 2 years in patients with axial spondyloarthritis (axSpA). In this post hoc analysis, we compare the impact of shorter versus longer symptom duration on the efficacy of bimekizumab to Week 104.

**Methods:**

Efficacy outcomes by symptom duration (≤ 2 [ASAS early axSpA definition] versus > 2 years; ≤ 5 versus > 5 years) were assessed across patients from BE MOBILE 1 and 2 (non-radiographic [NCT03928704]/radiographic axSpA [NCT03928743]) and the combined open-label extension (NCT04436640). (Relative) odds ratios and (relative) differences were calculated to compare 16-week bimekizumab versus placebo treatment effect and 104-week outcomes, and infer the significance of differences, between symptom duration subgroups. Analyses were neither powered for these comparisons nor multiplicity adjusted, and should be interpreted accordingly.

**Results:**

Improved disease activity, physical function, fatigue, health-related quality of life and objective signs of inflammation were seen with bimekizumab versus placebo at Week 16 regardless of symptom duration. Outcomes were then sustained or improved with bimekizumab to Week 104 across all subgroups.

16-week bimekizumab versus placebo treatment effect was comparable between subgroups (e.g., ≤ 2-year versus > 2-year symptom duration relative odds ratio [95% CI] for ASDAS < 2.1 in BE MOBILE 1: 0.82 [0.22, 3.08]). Significant differences were observed for some 104-week outcomes between symptom duration subgroups across both studies (e.g., ≤ 5-year versus > 5-year symptom duration odds ratio [95% CI] for ASDAS < 2.1 in BE MOBILE 2: 1.94 [1.02, 3.68]), all favouring the shorter symptom duration subgroups.

**Conclusions:**

Bimekizumab was efficacious to 2 years regardless of symptom duration, with comparable 16-week treatment effect but generally better 104-week outcomes in the shorter versus longer symptom duration subgroups.

**Trial registration:**

Registered on ClinicalTrials.gov; NCT03928704 (BE MOBILE 1; 23rd April 2019), NCT03928743 (BE MOBILE 2; 23rd April 2019), NCT04436640 (BE MOVING; 15th June 2020).

**Supplementary Information:**

The online version contains supplementary material available at 10.1186/s13075-026-03729-6.

## Introduction

Axial spondyloarthritis (axSpA), a chronic, immune-mediated inflammatory disease primarily affecting the axial skeleton, is characterised by back pain, spinal stiffness, and several peripheral and extra-musculoskeletal manifestations [1, 2]. Historically, axSpA has been split into two subtypes based on whether definitive evidence of structural damage is visible (radiographic [r-]axSpA) or not (non-radiographic [nr-]axSpA) on radiographs of sacroiliac joints (SIJ) [1, 2]. However, mounting evidence suggests nr-axSpA and r-axSpA are a continuum of one single disease [3, 4]. 

Patients with axSpA often experience major diagnostic delays, typically between 7 and 9 years after onset of chronic back pain [2]. Multiple factors contribute towards delays, including lack of disease awareness, absence of a gold-standard diagnosis tool, involvement of deep anatomical structures which are difficult to assess, and later presentation of specific clinical features such as evidence of damage on radiographs [2, 5]. As such, there is interest in understanding the pathophysiology and impact of treatment in early versus established disease stages of axSpA. Until recently, consensus on the definition of early axSpA was lacking, with a previous systematic literature review highlighting heterogeneous definitions of “early axSpA”[6]. Recognising the need for a uniform definition, in 2024, the Assessment of SpondyloArthritis international Society (ASAS) defined early axSpA for research purposes as duration of axial symptoms of ≤ 2 years [7]. 

Although numerous clinical trials have demonstrated the efficacy of biologic and targeted synthetic disease-modifying antirheumatic drugs (b/tsDMARDs) for the treatment of axSpA [8], there is currently limited research exploring whether treatment in early axSpA, using the ASAS consensus definition, leads to better outcomes than in established disease. Assessing the potential effects of early axSpA treatment on disease outcomes may provide insight on whether the window of opportunity concept (i.e., period potentially spanning months or years early in the disease course wherein treatment can enhance long-term prognostic outcomes) applies in axSpA, where research surrounding this concept is still limited [9]. 

Bimekizumab is a humanised monoclonal IgG1 antibody that selectively inhibits interleukin (IL)-17F in addition to IL-17A. Both cytokines are key mediators of inflammation that have been implicated in the pathogenesis of axSpA [10–12]. Bimekizumab 160 mg every 4 weeks (Q4W) has been approved for the treatment of axSpA by the European Medicines Agency and the United States Food and Drug Administration [13, 14]. Bimekizumab demonstrated sustained efficacy and was well tolerated up to 2 years across the full disease spectrum of axSpA in the phase 3 studies BE MOBILE 1 (nr-axSpA) and BE MOBILE 2 (r-axSpA), and their combined open-label extension (OLE), as well as up to 5 years in patients with r-axSpA in the phase 2b study BE AGILE [15–18]. 

Here, using the phase 3 data, we aimed to evaluate the impact of shorter versus longer symptom duration (≤ 2 versus > 2 years using the ASAS consensus definition of early axSpA, and ≤ 5 versus > 5 years as an exploratory grouping) on the efficacy of bimekizumab to 2 years of treatment across the full disease spectrum of axSpA.

## Methods

### Study design and oversight

The study design, inclusion and exclusion criteria for BE MOBILE 1 (NCT03928704) and BE MOBILE 2 (NCT03928743) have been previously described (Supplementary Figure S1) [15, 18]. Adult patients were eligible to enrol in the studies if they had active axSpA, defined as Bath Ankylosing Spondylitis Disease Activity Index (BASDAI) ≥ 4 and spinal pain (BASDAI question 2 [Q2]) ≥ 4. Patients in BE MOBILE 1 had nr-axSpA meeting ASAS classification criteria without definitive radiographic sacroiliitis and had objective inflammation at screening (active sacroiliitis on MRI and/or elevated C-reactive protein [CRP ≥ 6.0 mg/L]). Patients in BE MOBILE 2 had r-axSpA fulfilling the modified New York (mNY) criteria. All patients in BE MOBILE 2 also fulfilled ASAS criteria.

Both BE MOBILE studies comprised a 16-week double-blind placebo-controlled treatment period followed by a 36-week maintenance period. From Week 16, all patients received subcutaneous bimekizumab 160 mg Q4W to Week 52 [15, 18]. At Week 52, patients who completed either study without meeting any withdrawal criteria were eligible to enrol in the OLE (BE MOVING; NCT04436640), where all patients continued to receive bimekizumab (Supplementary Figure S1).

### Outcomes

This post hoc analysis aimed to report measures of disease activity, physical function, fatigue, health-related quality of life (HRQoL) and objective signs of inflammation to Week 104 (i.e., up to 2 years of total treatment duration) for patients with shorter versus longer symptom duration (≤ 2 versus > 2 years and ≤ 5 versus > 5 years) in BE MOBILE 1 and BE MOBILE 2. In BE MOBILE 1, patients were stratified based on a symptom duration of ≤ 2 or > 2 years per ASAS consensus definition [7], but not in BE MOBILE 2 due to low patient numbers in the ≤ 2-year symptom duration subgroup. An additional stratification of ≤ 5 or > 5 years was used as this threshold was close to the median and first quartile for symptom duration in BE MOBILE 1 and 2, respectively. For all outcomes, results from bimekizumab-randomised patients and placebo-randomised patients who switched to bimekizumab were pooled within each study from Week 52.

Response to treatment was assessed based on proportion of patients achieving ASAS 40% response (ASAS40), Axial Spondyloarthritis Disease Activity Score (ASDAS) outcomes (ASDAS < 2.1, ASDAS < 1.3, ASDAS clinically important improvement [CII; ≥ 1.1-point reduction from baseline] and ASDAS major improvement [MI; ≥ 2.0-point reduction from baseline]) and mean change from baseline (CfB) in BASDAI score. Details on disease activity outcomes have been previously reported [17, 18]. 

Physical function was assessed using mean CfB in Bath Ankylosing Spondylitis Functional Index (BASFI), which assesses functional anatomical limitations and the ability to perform daily activities, with a scoring range of 0–10; lower scores indicate better physical function.

Fatigue was assessed using mean CfB in BASDAI Q1, which was assessed on a 0–10 numerical rating scale; higher scores reflect more fatigue.

HRQoL was assessed using mean CfB in Ankylosing Spondylitis Quality of Life (ASQoL) questionnaire scores, an 18-item questionnaire assessing the impact of axSpA on patient HRQoL, where an item score of “1” represents a patient responding “yes” and “0” representing a “no”. The ASQoL score is obtained by summing each item score which ranges from 0 to 18, with higher scores indicating worse HRQoL [19]. 

For objective signs of inflammation, mean CfB in MRI Spondyloarthritis Research Consortium of Canada (SPARCC) SIJ inflammation score (total score ranging between 0 and 72; nr-axSpA only [BE MOBILE 1]) and Berlin modification of the spine ASspiMRI-a score (hereafter termed MRI Berlin spine score with total score ranging between 0 and 69; r-axSpA only [BE MOBILE 2]) were assessed out to 16 and 104 weeks in two separate reading campaigns in the subset of patients enrolled in the MRI sub-studies [17, 18]. Objective signs of inflammation were also assessed using the mean CfB in high-sensitivity CRP (hs-CRP) values.

### Statistical analysis

All outcomes in this post hoc analysis were summarised by treatment group and stratified by symptom duration (≤ 2 versus > 2 years and ≤ 5 versus > 5 years) for randomised populations in BE MOBILE 1 and 2. All analyses were performed on the randomised set (i.e., all patients randomised in BE MOBILE 1 and 2).

All ASDAS, BASDAI, BASFI, ASQoL and hs-CRP outcomes were imputed using multiple imputation (MI); ASDAS status, ASDAS-CII and ASDAS-MI were derived from the imputed ASDAS. The proportion of patients achieving ASAS40 was imputed using non-responder imputation (NRI). All MRI inflammation outcomes were not imputed and are presented using observed case (OC).

To assess potential differences in 16-week treatment effect of bimekizumab versus placebo and 104-week outcomes between symptom duration subgroups, Week 16 relative odds ratios and Week 104 odds ratios for binary outcomes were calculated using logistic regression, and Week 16 relative differences and Week 104 differences in least-square means (LSM) of continuous outcomes were calculated using analysis of covariance (ANCOVA). Week 104 odds ratios and differences were calculated using 104-week outcomes from pooled placebo- and bimekizumab-randomised patients.

Factors for logistic regression and ANCOVA at Week 16 included treatment, region, baseline value for the respective continuous variable (ANCOVA only), symptom duration and the interaction between treatment and symptom duration (treatment × symptom duration). These models were additionally adjusted for MRI/CRP classification and prior tumour necrosis factor inhibitor (TNFi) exposure in patients enrolled in BE MOBILE 1 and 2, respectively. Factors for logistic regression and ANCOVA at Week 104 included region, baseline value for the respective continuous variable (ANCOVA only) and symptom duration.

A (relative) odds ratio > 1 or a (relative) difference < 0 indicates better efficacy (16-week bimekizumab versus placebo treatment effect, or 104-week outcomes) in the shorter (≤ 2/≤ 5-year) versus longer (> 2/> 5-year) symptom duration subgroup, and vice versa. These thresholds were used to determine whether there were ‘significant differences’ in efficacy between the subgroups, while recognising the need for careful interpretation given these post hoc analyses were not powered specifically for this, nor were they adjusted for multiplicity. Comparisons of 16-week bimekizumab versus placebo treatment effect take patient response to placebo into account, while comparisons of 104-week outcomes are performed in bimekizumab-treated patients with no placebo comparator.

## Results

**Fig. 1:**
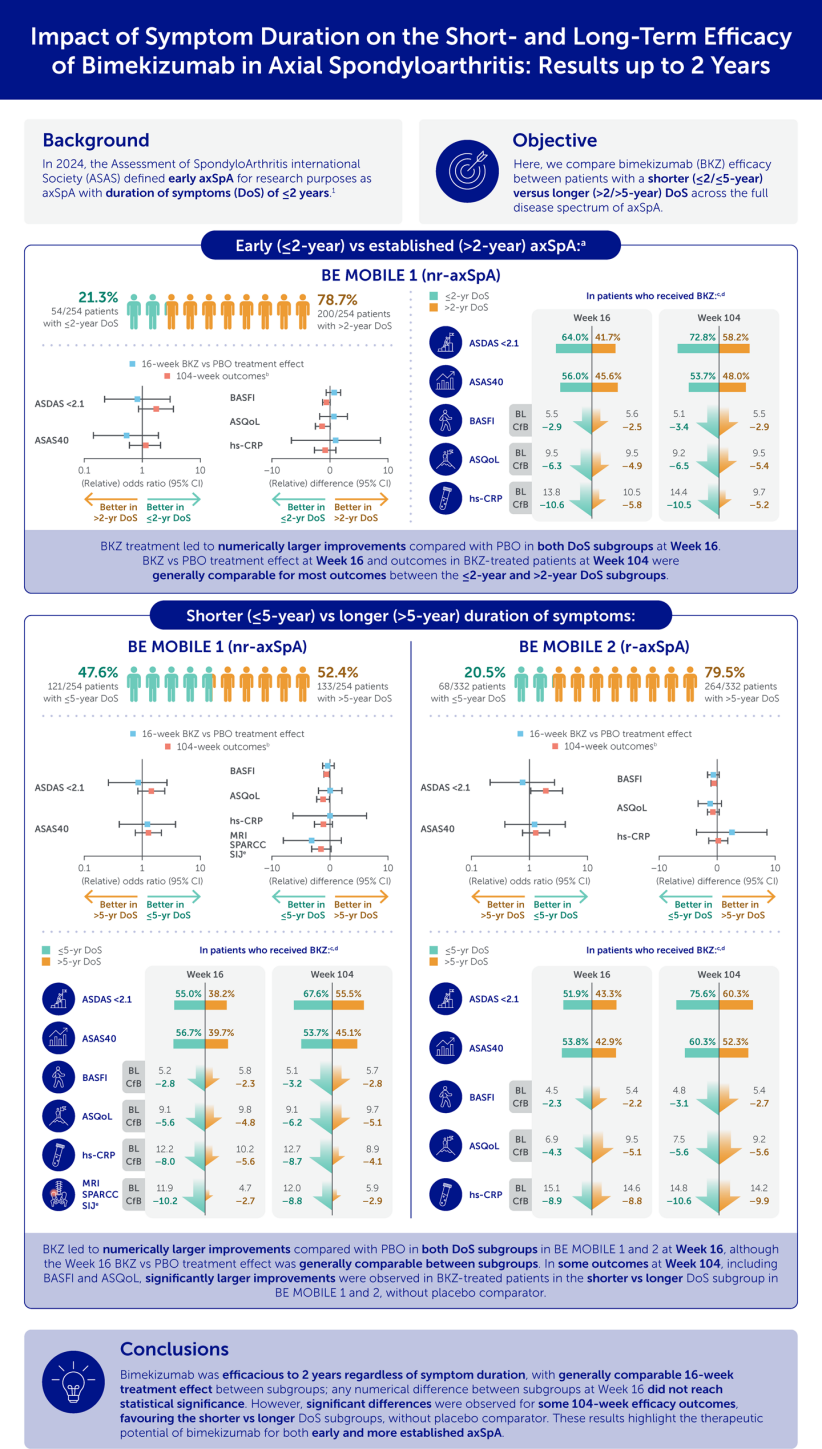
Graphical summary of study results. 1. Navarro-Compán V. Ann Rheum Dis 2024;83:1093–9. ^a^Only investigated in BE MOBILE 1 due to low patient numbers with early axSpA in BE MOBILE 2; ^b^In pooled BKZ- and PBO-randomised patients; ^c^In BKZ-randomised patients only at Week 16 and in pooled BKZ- and PBO-randomised patients at Week 104; ^d^Continuous variables (BASFI, ASQoL, hs-CRP and MRI SPARCC SIJ) are presented as the mean absolute value at baseline and mean change from baseline at each timepoint; ^e^Only investigated for ≤ 5/> 5-year DoS due to low numbers of patients with early axSpA who were enrolled in the MRI sub-study. ASAS: Assessment of SpondyloArthritis international Society; ASAS40: ASAS 40% response; ASDAS: Axial Spondyloarthritis Disease Activity Score; ASQoL: Ankylosing Spondylitis Quality of Life questionnaire; axSpA: axial spondyloarthritis; BASFI: Bath Ankylosing Spondylitis Functional Index; BKZ: bimekizumab; BL: baseline; CfB: change from baseline; CI: confidence interval; DoS: duration of symptoms; hs-CRP: high-sensitivity C-reactive protein; MRI: magnetic resonance imaging; nr-axSpA: non-radiographic axSpA; PBO: placebo; r-axSpA: radiographic axSpA; SIJ: sacroiliac joint; SPARCC: Spondyloarthritis Research Consortium of Canada; yr: year

A graphical summary of key outcomes from this post hoc analysis is presented in Fig. 1.

### Patient disposition and baseline characteristics

Of the 586 patients randomised in BE MOBILE 1 and 2 (254 and 332 patients, respectively), 494/586 (84.3%) entered the OLE at Week 52, with 456 patients completing Week 104 (77.8% of randomised patients and 92.3% of patients entering the OLE) at the time of the data-cut on 6th July 2023 (Supplementary Figure S2) [17]. 

In BE MOBILE 1, patients were categorised into the ≤ 2-year (54/254 [21.3%]) and > 2-year (200/254 [78.7%]) symptom duration subgroups, as well as the ≤ 5-year (121/254 [47.6%]) and > 5-year (133/254 [52.4%]) symptom duration subgroups. In BE MOBILE 2, patients were categorised into the ≤ 5-year (68/332 [20.5%]) versus > 5-year (264/332 [79.5%]) symptom duration subgroups. Due to low patient numbers in the ≤ 2-year symptom duration subgroups for clinical efficacy outcomes in BE MOBILE 2 patients (17/332 [5.1%]), and for MRI outcomes in BE MOBILE 1 (MRI SPARCC SIJ; 31/152 [20.4%]) and BE MOBILE 2 patients (MRI Berlin spine; 7/137 [5.1%]), it was not possible to evaluate these outcomes in the ≤ 2-year versus > 2-year symptom duration subgroups. Additionally, MRI Berlin spine scores were not evaluated in the ≤ 5-year and > 5-year symptom duration subgroups in BE MOBILE 2 due to comparatively low patient numbers in the ≤ 5-year (26/137 [19.0%]) versus the > 5-year (111/137 [81.0%]) symptom duration subgroup.

Patient demographics and baseline characteristics were largely similar between symptom duration subgroups and their respective overall study populations (Table 1). However, at baseline, patients in the ≤ 2-year and ≤ 5-year symptom duration subgroups on average were younger, had lower BMI and showed higher MRI SPARCC SIJ inflammation, and a larger proportion of patients were male and HLA-B27 positive. Conversely, a larger proportion of patients in the > 2-year and > 5-year symptom duration subgroups had previous exposure to TNFis.

**Table 1:** Patient demographics and baseline characteristics, stratified by symptom duration

	BE MOBILE 1 (nr-axSpA)					BE MOBILE 2 (r-axSpA)		
Mean (SD), unless otherwise stated	≤ 2-year DoS (*N* = 54)	> 2-year DoS (*N* = 200)	≤ 5-year DoS (*N* = 121)	> 5-year DoS (*N* = 133)	Overall study population (*N* = 254)	≤ 5-year DoS (*N* = 68)	> 5-year DoS (*N* = 264)	Overall study population (*N* = 332)
**Patient demographics**								
Age, years	31.7 (7.0)	41.5 (11.5)	32.2 (7.7)	46.0 (10.3)	39.4 (11.5)	30.8 (8.4)	42.9 (11.9)	40.4 (12.3)
Sex, male, *n* (%)	37 (68.5)	101 (50.5)	75 (62.0)	63 (47.4)	138 (54.3)	52 (76.5)	188 (71.2)	240 (72.3)
Geographical region,^a^ *n* (%)								
Asia^b^	11 (20.4)	17 (8.5)	17 (14.0)	11 (8.3)	28 (11.0)	18 (26.5)	43 (16.3)	61 (18.4)
Eastern Europe^c^	35 (64.8)	109 (54.5)	76 (62.8)	68 (51.1)	144 (56.7)	38 (55.9)	125 (47.3)	163 (49.1)
Western Europe^d^	7 (13.0)	57 (28.5)	25 (20.7)	39 (29.3)	64 (25.2)	12 (17.6)	87 (33.0)	99 (29.8)
North America^e^	1 (1.9)	17 (8.5)	3 (2.5)	15 (11.3)	18 (7.1)	0	9 (3.4)	9 (2.7)
BMI, kg/m^2^	25.7 (4.7)	27.9 (6.0)	26.3 (5.2)	28.4 (6.2)	27.4 (5.8)	25.0 (5.0)	27.3 (5.8)	26.9 (5.8)
Time since first symptoms of axSpA, years	1.2 (0.5)	11.1 (8.8)	2.4 (1.3)	15.0 (8.5)	9.0 (8.8)	3.0 (1.3)	16.2 (9.9)	13.5 (10.3)
**Disease characteristics**								
HLA-B27 positive, *n* (%)	44 (81.5)	153 (76.5)	99 (81.8)	98 (73.7)	197 (77.6)	58 (85.3)	226 (85.6)	284 (85.5)
hs-CRP, mg/L	14.4 (16.2)	9.7 (12.3)	12.7 (15.4)	8.9 (10.9)	10.7 (13.3)	14.8 (21.4)	14.2 (16.7)	14.3 (17.7)
ASDAS	3.7 (0.8)	3.7 (0.7)	3.7 (0.8)	3.7 (0.7)	3.7 (0.7)	3.6 (0.8)	3.7 (0.8)	3.7 (0.8)^f^
BASDAI (0–10)	6.4 (1.2)	6.9 (1.3)	6.6 (1.3)	7.0 (1.2)	6.8 (1.3)	6.2 (1.4)	6.5 (1.3)	6.5 (1.3)
BASFI^g^ (0–10)	5.1 (2.4)	5.5 (2.2)	5.1 (2.4)	5.7 (2.1)	5.4 (2.3)	4.8 (2.4)	5.4 (2.0)	5.2 (2.1)
BASDAI Q1 (0–10)	6.1 (1.6)	6.7 (1.7)	6.4 (1.6)	6.7 (1.7)	6.6 (1.7)	6.2 (1.4)	6.5 (1.6)	6.4 (1.5)
ASQoL (0–18)	9.2 (4.7)	9.5 (4.4)	9.1 (4.6)	9.7 (4.4)	9.4 (4.5)	7.5 (4.8)	9.2 (4.5)	8.9 (4.6)
MRI Berlin spine score^h^ (0–69)	–	–	–	–	–	2.2 (3.7)^i^	3.4 (4.5)^j^	3.2 (4.4)^k^
MRI SPARCC SIJ score^h^ (0–72)	12.9 (13.1)^l^	7.8 (10.5)^m^	12.0 (12.5)^n^	5.9 (9.1)^o^	8.8 (11.3)^p^	–	–	–
EMM history,^q^ *n* (%)								
IBD	0	4 (2.0)	1 (0.8)	3 (2.3)	4 (1.6)	0	4 (1.5)	4 (1.2)
Uveitis	5 (9.3)	35 (17.5)	16 (13.2)	24 (18.0)	40 (15.7)	8 (11.8)	49 (18.6)	57 (17.2)
Psoriasis	1 (1.9)	15 (7.5)	7 (5.8)	9 (6.8)	16 (6.3)	5 (7.4)	21 (8.0)	26 (7.8)
Prior TNFi exposure (TNFi-IR patients),^r^ *n* (%)	2 (3.7)	25 (12.5)	8 (6.6)	19 (14.3)	27 (10.6)	7 (10.3)	47 (17.8)	54 (16.3)
Concomitant medication use at baseline, *n* (%)								
NSAIDs	43 (79.6)	146 (73.0)	93 (76.9)	96 (72.2)	189 (74.4)	55 (80.9)	211 (79.9)	266 (80.1)
Oral glucocorticoids	7 (13.0)	14 (7.0)	8 (6.6)	13 (9.8)	21 (8.3)	3 (4.4)	20 (7.6)	23 (6.9)
csDMARDs	15 (27.8)	46 (23.0)	27 (22.3)	34 (25.6)	61 (24.0)^s^	13 (19.1)	53 (20.1)	66 (19.9)^s^

Randomised sets. ^a^Patients categorised by the stratum to which they were randomised; ^b^Includes China, Japan and Turkey; ^c^Includes Bulgaria, the Czech Republic, Hungary and Poland; ^d^Includes Belgium, France, Germany, The Netherlands, Spain and the United Kingdom; ^e^Includes United States of America only; ^f^*n*=331; ^g^Individual component of the primary outcome measure (ASAS40); ^h^In patients in the MRI sub-study; ^i^*n*=26; ^j^*n*=111; ^k^*n*=137; ^l^*n*=31; ^m^*n*=121; ^n^*n*=73; ^o^*n*=79; ^p^*n*=152; ^q^Based on extra-musculoskeletal assessments at screening or baseline; ^r^Defined as patients who were intolerant or experienced an inadequate response to previous TNFi treatment given at an approved dose for at least 12 weeks; ^s^Includes methotrexate in 21 patients with nr-axSpA and 12 patients with r-axSpA, sulfasalazine in 33 patients with nr-axSpA and 52 patients with r-axSpA. ASAS40: Assessment of SpondylArthritis international Society 40% response; ASDAS: Axial Spondyloarthritis Disease Activity Score; ASQoL: Ankylosing Spondylitis Quality of Life questionnaire; axSpA: axial spondyloarthritis; BASDAI: Bath Ankylosing Spondylitis Disease Activity Index; BASFI: Bath Ankylosing Spondylitis Functional Index; csDMARD: conventional synthetic disease-modifying antirheumatic drug; DoS: duration of symptoms; EMM: extra-musculoskeletal manifestation; HLA‑B27: human leukocyte antigen B27; hs‑CRP: high sensitivity C‑reactive protein; IBD: inflammatory bowel disease; NSAID: nonsteroidal anti-inflammatory drug; MRI: magnetic resonance imaging; nr-axSpA: non-radiographic axial spondyloarthritis; r-axSpA: radiographic axial spondyloarthritis; SD: standard deviation; TNFi: tumour necrosis factor inhibitor

### Efficacy in early (≤ 2-year) versus established (> 2-year symptom duration) axSpA (BE MOBILE 1; nr-axSpA)

**Fig. 2:**
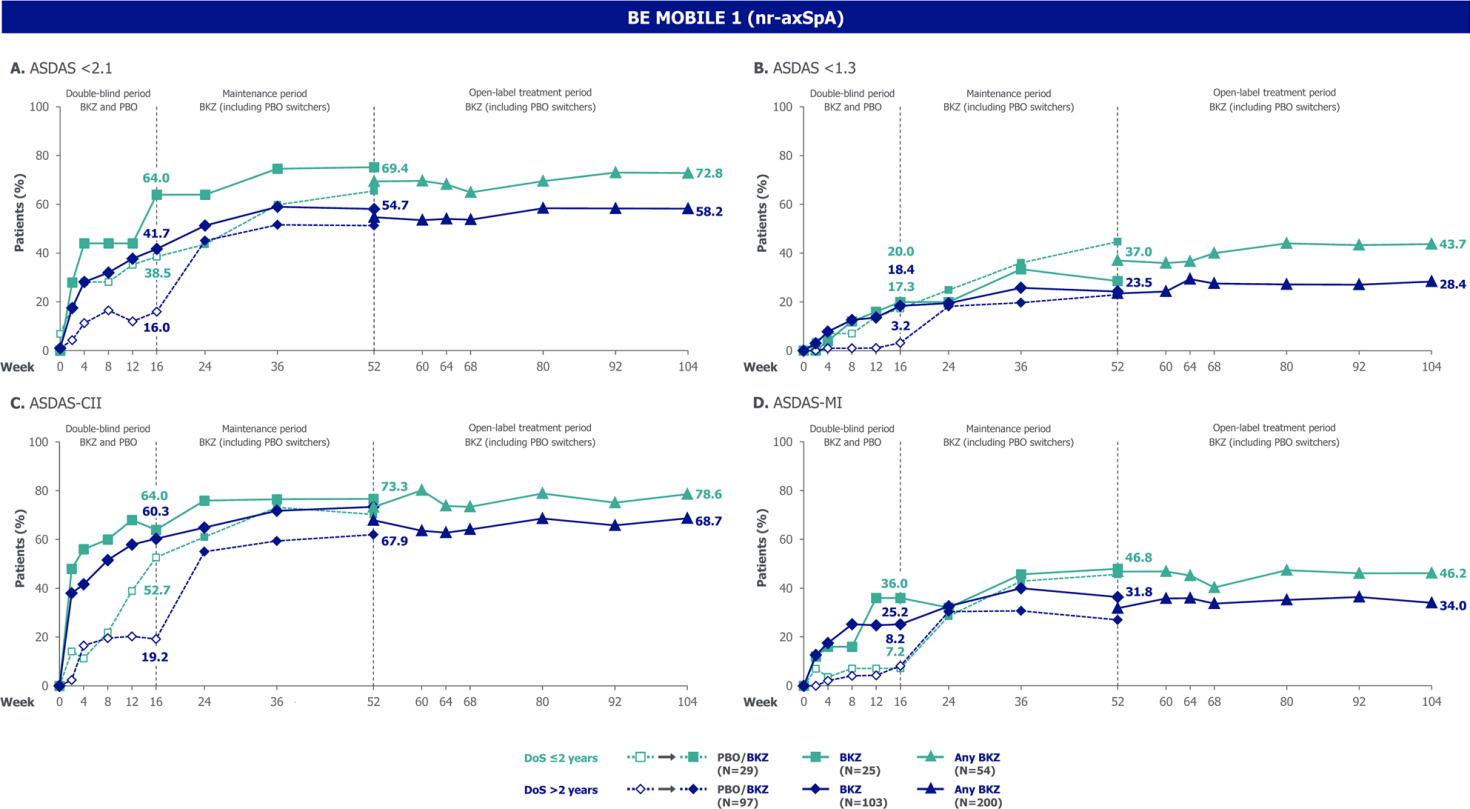
ASDAS outcomes stratified by symptom duration ≤ 2 and > 2 years in BE MOBILE 1 (MI). Randomised set. N refers to the number of patients in their respective subgroups at baseline. Beyond Week 52, all data are shown pooled across bimekizumab- and placebo-randomised patients. ASDAS: Axial Spondyloarthritis Disease Activity Score; ASDAS-CII: ASDAS clinically important improvement; ASDAS-MI: ASDAS major improvement; axSpA: axial spondyloarthritis; BKZ: bimekizumab; DoS: duration of symptoms; MI: multiple imputation; nr-axSpA: non-radiographic axial spondyloarthritis; PBO: placebo

**Fig. 3:**
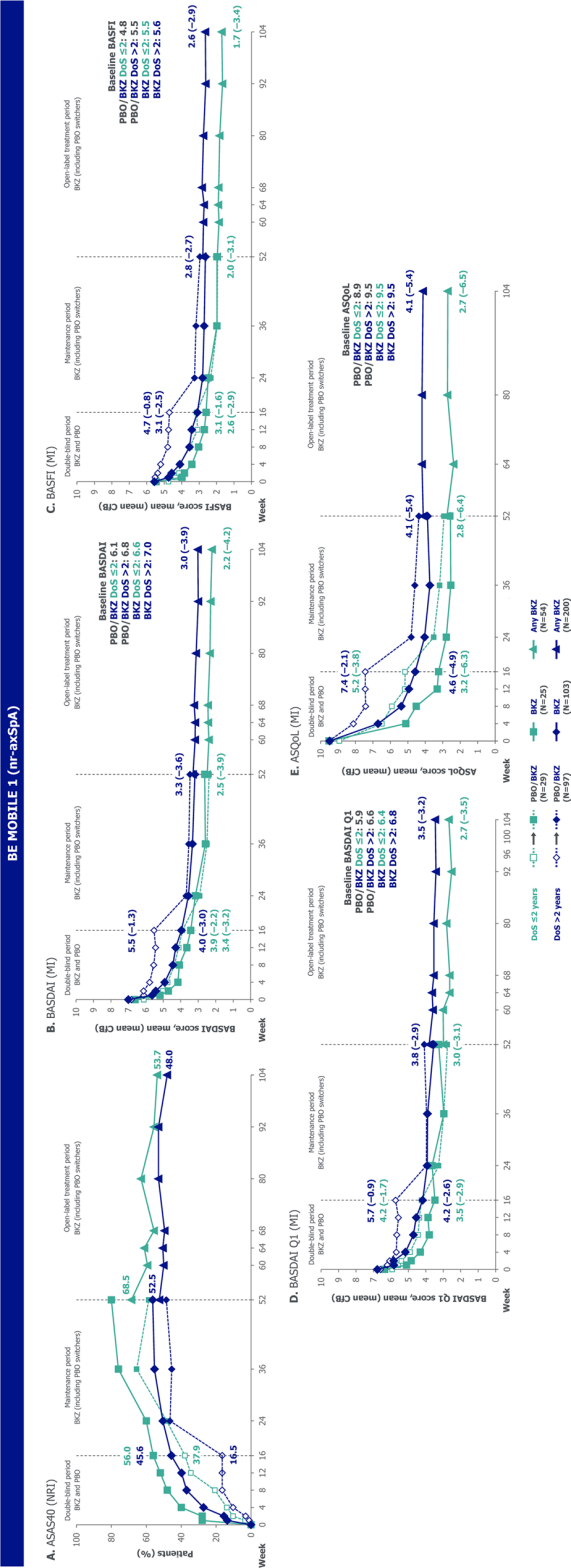
Key efficacy outcomes stratified by symptom duration ≤ 2 and > 2 years in BE MOBILE 1. Randomised set. N refers to the number of patients in their respective subgroups at baseline. Beyond Week 52, all data are pooled across bimekizumab- and placebo-randomised patients. ASAS40: Assessment of SpondyloArthritis international Society 40% response; ASQoL: Ankylosing Spondylitis Quality of Life questionnaire; axSpA: axial spondyloarthritis; BASDAI: Bath Ankylosing Spondylitis Disease Activity Index; BASFI: Bath Ankylosing Spondylitis Functional Index; BKZ: bimekizumab; CfB: change from baseline; DoS: duration of symptoms; MI: multiple imputation; nr-axSpA: non-radiographic axial spondyloarthritis; NRI: non-responder imputation; PBO: placebo

**Fig. 4:**
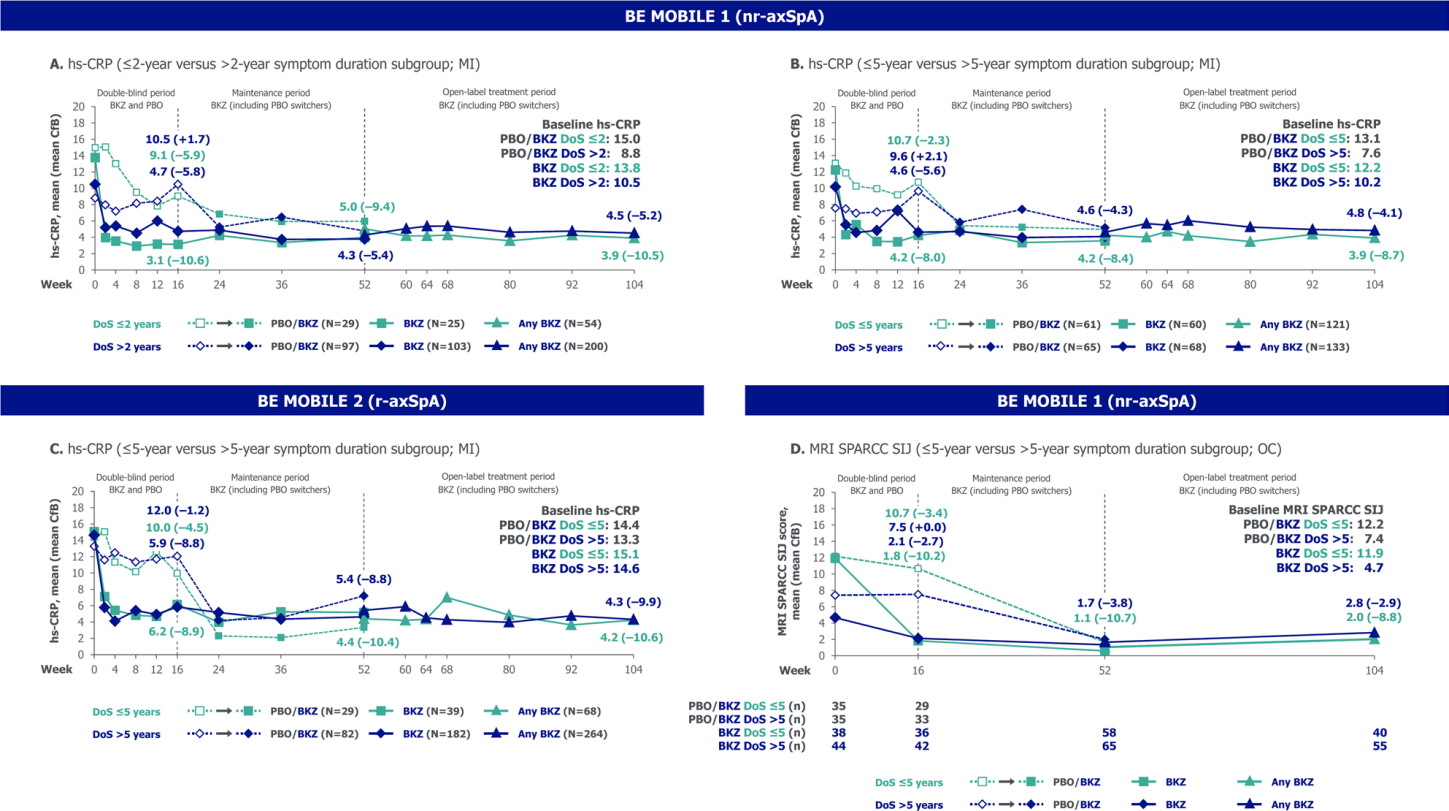
Objective signs of inflammation stratified by symptom duration in BE MOBILE 1 and 2. Randomised sets. For MRI SPARCC SIJ outcomes, only study participants enrolled in the MRI sub-studies are included. Beyond Week 52, all data, including n numbers, are pooled across BKZ- and PBO-randomised patients. axSpA: axial spondyloarthritis; BKZ: bimekizumab; CfB: change from baseline; DoS: duration of symptoms; hs-CRP: high sensitivity C-reactive protein; MI: multiple imputation; MRI: magnetic resonance imaging; nr-axSpA: non-radiographic axial spondyloarthritis; OC: observed case; PBO: placebo; r-axSpA; radiographic axial spondyloarthritis; SIJ: sacroiliac joint; SPARCC: Spondyloarthritis Research Consortium of Canada

Within each subgroup, outcomes measuring disease activity, physical function, fatigue, HRQoL and objective signs of inflammation demonstrated the overall efficacy of bimekizumab versus placebo to Week 16 (Figs. 2, 3 and 4). After Week 16, within each subgroup, outcomes of patients randomised to bimekizumab were maintained or further improved to Week 52; these positive outcomes were matched at Week 52 by placebo-randomised patients after they switched to bimekizumab at Week 16. Outcomes were then generally sustained to Week 104.

**Fig. 5:**
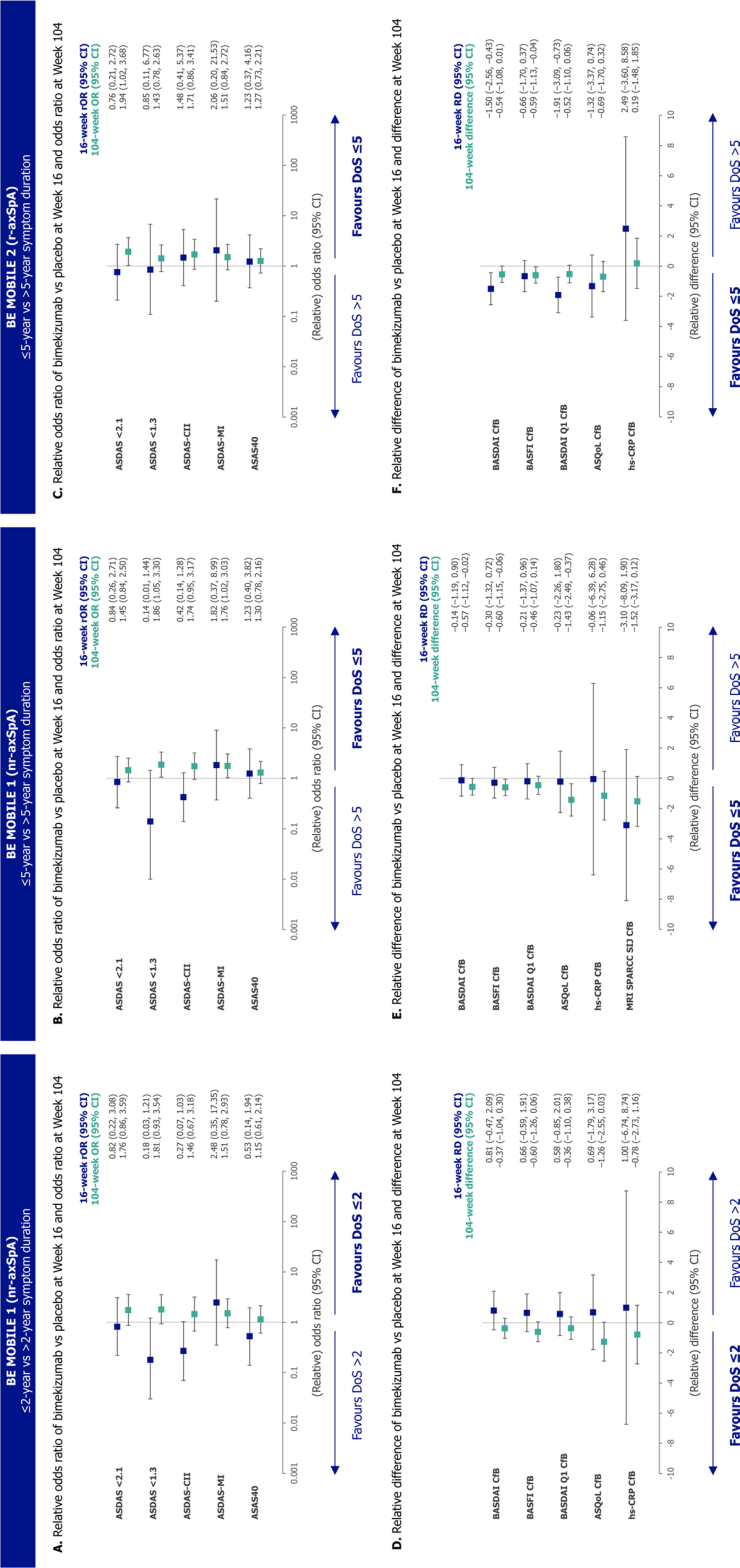
Comparison of 16-week bimekizumab versus placebo treatment effect and 104-week outcomes between symptom duration subgroups. Randomised sets. A (relative) odds ratio > 1 or a (relative) difference < 0 indicates better efficacy (16-week bimekizumab versus placebo treatment effect, or 104-week outcomes) in the shorter symptom duration subgroup compared with the longer symptom duration subgroup, and vice versa. ASAS40: Assessment of SpondyloArthritis international Society 40% response; ASDAS: Axial Spondyloarthritis Disease Activity Score; ASDAS-CII: ASDAS clinically important improvement; ASDAS-MI: ASDAS major improvement; ASQoL: Ankylosing Spondylitis Quality of Life questionnaire; axSpA: axial spondyloarthritis; BASDAI: Bath Ankylosing Spondylitis Disease Activity Index; BASFI: Bath Ankylosing Spondylitis Functional Index; CfB: change from baseline; CI: confidence interval; hs-CRP: high sensitivity C-reactive protein; MRI: magnetic resonance imaging; nr-axSpA: non-radiographic axial spondyloarthritis; OR: odds ratio; Q1: question one; r-axSpA: radiographic axial spondyloarthritis; RD: relative difference; rOR: relative odds ratio; SIJ: sacroiliac joint; SPARCC: Spondyloarthritis Research Consortium of Canada

Comparing subgroups within each treatment group, outcomes were generally numerically better in the ≤ 2 versus > 2-year symptom duration subgroup throughout the 104 weeks (Figs. 2, 3 and 4). 16-week bimekizumab versus placebo treatment effect was comparable or numerically larger, without reaching significant difference, in the > 2-year versus ≤ 2-year symptom duration subgroup. In contrast, the opposite trend was observed for 104-week outcomes (without placebo comparator) favouring patients with a shorter symptom duration, but still without reaching significant difference (Fig. 5).

For example, a numerically higher proportion of patients in the ≤ 2-year versus > 2-year symptom duration subgroup achieved ASDAS < 2.1 at Week 16 (bimekizumab: 64.0% versus 41.7%; placebo: 38.5% versus 16.0%; Fig. 2A). However, for ASDAS < 2.1, the 16-week bimekizumab versus placebo treatment effect was comparable between subgroups (relative odds ratio [95% CI]: 0.82 [0.22, 3.08]; Fig. 5A). The proportion of patients achieving ASDAS < 2.1 continued to improve to Week 104 with bimekizumab treatment across both subgroups, and was numerically higher, but not significantly different, in the ≤ 2-year versus > 2-year symptom duration subgroup (Week 104: 72.8% versus 58.2%; odds ratio [95% CI]: 1.76 [0.86, 3.59]).

### Efficacy in shorter (≤ 5-year) versus longer (> 5-year) symptom duration subgroups (BE MOBILE 1 and 2; nr- and r-axSpA)

**Fig. 6:**
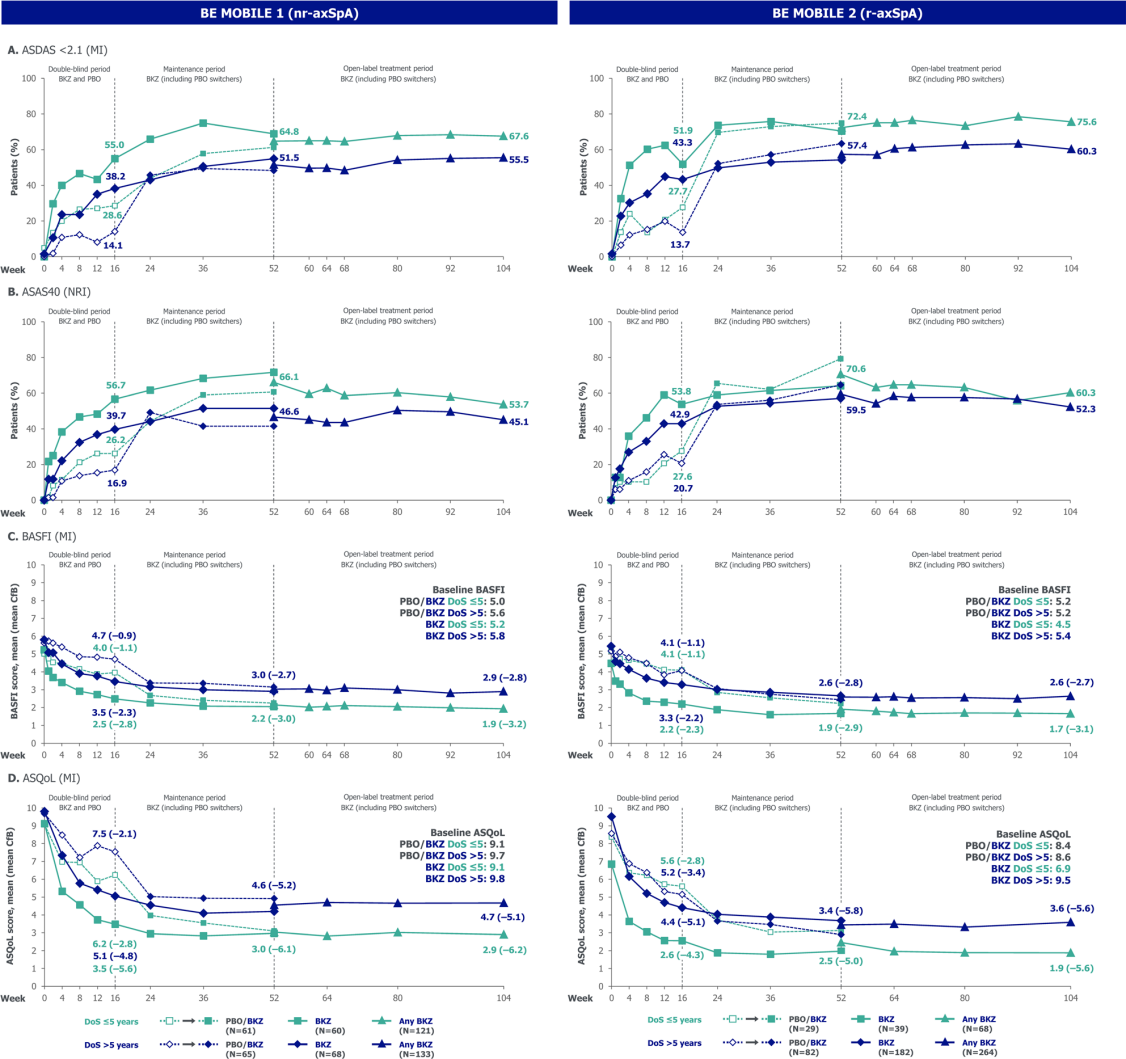
Outcomes stratified by symptom duration ≤ 5 and > 5 years in BE MOBILE 1 and 2. Randomised sets. N refers to the number of patients in their respective subgroups at baseline. Beyond Week 52, all data are pooled across bimekizumab- and placebo-randomised patients. ASAS40: Assessment of SpondyloArthritis international Society 40% response; ASDAS: Axial Spondyloarthritis Disease Activity Score; ASQoL: Ankylosing Spondylitis Quality of Life questionnaire; axSpA: axial spondyloarthritis; BASDAI: Bath Ankylosing Spondylitis Disease Activity Index; BASFI: Bath Ankylosing Spondylitis Functional Index; BKZ: bimekizumab; CfB: change from baseline; DoS: duration of symptoms; MI: multiple imputation; nr-axSpA: non-radiographic axial spondyloarthritis; NRI: non-responder imputation; PBO: placebo; Q1: question one; r-axSpA: radiographic axial spondyloarthritis

Across both the ≤ 5-year and > 5-year symptom duration subgroups in BE MOBILE 1 and 2 (Figs. 4 and 6, Supplementary Figure S3), the pattern of improvement over time within each subgroup and comparisons between subgroups were similar to those observed in the ≤ 2-year versus > 2-year symptom duration subgroups. The relative odds ratio and relative difference analysis showed that 16-week bimekizumab versus placebo treatment effect was generally comparable or numerically larger, without reaching significance, in the ≤ 5-year versus > 5-year symptom duration subgroup across the full disease spectrum of axSpA (Fig. 5). Significant differences between subgroups in 16-week bimekizumab versus placebo treatment effect were only detected in BE MOBILE 2 for BASDAI CfB and BASDAI Q1 CfB, where treatment effect was larger in the ≤ 5-year symptom duration subgroup. Conversely, significant differences between subgroups were observed in more 104-week outcomes (without placebo comparator) across both studies (BE MOBILE 1: ASDAS < 1.3, ASDAS-MI, BASDAI CfB, BASFI CfB and ASQoL CfB; BE MOBILE 2: ASDAS < 2.1 and BASFI CfB), all demonstrating better outcomes in the ≤ 5-year symptom duration subgroup.

For example, in BE MOBILE 1, a numerically larger proportion of patients in the ≤ 5-year versus > 5-year symptom duration subgroup achieved ASDAS < 2.1 at Week 16 (bimekizumab: 55.0% versus 38.2%; placebo: 28.6% versus 14.1%). However, 16-week bimekizumab versus placebo treatment effect on the achievement of ASDAS < 2.1 was comparable between subgroups (relative odds ratio [95% CI]: 0.84 [0.26, 2.71]; Fig. 5B). The proportion of patients achieving ASDAS < 2.1 continued to improve to Week 104 with bimekizumab treatment across both subgroups, and was numerically higher, but not significantly different, in the ≤ 5-year versus > 5-year symptom duration subgroup (Week 104: 67.6% versus 55.5%; odds ratio [95% CI]: 1.45 [0.84, 2.50]). Similar ASDAS < 2.1 results were also observed in BE MOBILE 2, although a significant difference in the proportion of patients achieving ASDAS < 2.1 was observed at Week 104 between subgroups (odds ratio [95% CI]: 1.94 [1.02, 3.68]), without placebo comparator (Fig. 5C).

In the BE MOBILE 1 MRI sub-study, 152 patients had an MRI SPARCC SIJ assessment at baseline (total n by symptom duration subgroup: ≤ 5-year: 73; > 5-year: 79). At baseline, mean MRI SPARCC SIJ scores were numerically higher in the ≤ 5-year versus > 5-year symptom duration subgroup in BE MOBILE 1 (Fig. 4D). At Week 16, a numerically larger reduction from baseline in mean MRI SPARCC SIJ score was observed in the ≤ 5-year versus > 5-year symptom duration subgroup; improvements were largely maintained to Week 104. 16-week bimekizumab versus placebo treatment effect and 104-week outcomes (without placebo comparator) for MRI SPARCC SIJ CfB were numerically larger, but not significantly different in the ≤ 5-year versus > 5-year symptom duration subgroup.

## Discussion

Using the ASAS definition of early axSpA (≤ 2-year symptom duration) and an additional symptom duration threshold (≤ 5-year symptom duration), this post hoc analysis expands on published long-term efficacy of bimekizumab to address the evidence gap on the potential impact of earlier versus later treatment for axSpA [7, 17]. Across disease activity, physical function, fatigue, HRQoL and objective signs of inflammation outcomes, our findings demonstrated the sustained efficacy of bimekizumab to Week 104 regardless of symptom duration. We did not find significant differences in treatment effect at Week 16 between shorter and longer symptom duration subgroups when taking bimekizumab and placebo response into account, although within each treatment arm there was a numerically higher response in the shorter symptom duration subgroups. It should be noted that, since this post-hoc analysis was not sufficiently powered for statistical comparisons, significant differences may possibly emerge for these outcomes should the analyses be repeated using larger sample sizes. While significantly better outcomes were observed at Week 104 in the shorter versus longer symptom duration subgroup, differences cannot be definitively attributed to bimekizumab given the absence of a placebo comparator group after Week 16.

These findings, which show that bimekizumab treatment is efficacious regardless of symptom duration, align with previous observational, randomised controlled and meta-analysis studies covering a range of b/tsDMARDs that found axSpA treatment to be equally efficacious in patients grouped using a ≤ 2-year and > 2-year symptom duration threshold [20–22]. However, to our knowledge, no symptom duration subgroup analyses on IL-17A inhibitors have been published.

Different criteria have been used in past literature to define early axSpA, including disease duration (i.e., time since diagnosis) and absence or presence of radiographic damage [23]. These included randomised controlled studies on the IL-17A inhibitors secukinumab and ixekizumab, which defined early axSpA based on disease duration [24–26]. When early axSpA was defined by disease duration or radiographic damage, a systematic literature review found no significant differences in outcomes between early and established axSpA. However, the same review found that when early axSpA was defined as a symptom duration of < 5 years, early treatment was associated with improved outcomes, although evidence was limited and only found in two nr-axSpA studies [23]. 

When assessing objective signs of inflammation, patients with nr-axSpA in our ≤ 5-year versus > 5-year symptom duration subgroup exhibited higher baseline MRI SIJ inflammation. These results suggest that higher inflammatory burden of the SIJ may be present in earlier axSpA stages, aligning with the current clinical approach of utilising MRI examination of the SIJ, rather than the spine, as a diagnostic tool for axSpA [2]. However, due to low patient numbers with MRI assessments in the ≤ 2-year and ≤ 5-year symptom duration subgroup, further clinical evidence is required to elucidate the development of SIJ and spine inflammation throughout the disease progression of axSpA and the potential benefits of early treatment.

A key strength of this study is that it evaluated long-term bimekizumab treatment across the full disease spectrum of axSpA. This study utilised the consensus definition of early axSpA recently established by ASAS, contributing to the growing literature using that definition and supporting its consistent use across emerging research on axSpA treatment [7]. In addition, the use of relative odds ratio and relative difference analyses allowed for a comparison of the efficacy of bimekizumab between the shorter and longer symptom duration subgroups while accounting for patient response to placebo, helping to determine if observed differences were due to the treatment effect of bimekizumab or also seen in placebo-treated patients.

However, the treatment effect of bimekizumab compared with placebo could not be investigated at Week 104 due to a lack of placebo arm after Week 16. Additionally, due to the limited patient numbers in the ≤ 2-year symptom duration subgroups in BE MOBILE 2, subgroup analyses in that study were only possible using ≤ 5-year and > 5-year symptom duration subgroups and not those based on the ASAS consensus definition for early axSpA. Limited patient numbers in the ≤ 2-year symptom duration subgroup in BE MOBILE 2 could suggest that patients with r-axSpA generally have a longer symptom duration compared with patients with nr-axSpA, as observed in the BE MOBILE 2 versus BE MOBILE 1 study (13.5 versus 9.0 years at baseline). However, based on clinical expert consensus, the ASAS working group agreed that involvement of radiographic damage should not be taken into account when defining early axSpA, thereby allowing patients with radiographic sacroiliitis (i.e., r-axSpA) to meet the criteria for early axSpA when axial symptoms started less than two years ago [7]. Another limitation mentioned previously was that, due to the post hoc nature of the analysis and low patient numbers in some subgroups, the analyses lacked sufficient power for statistical significance testing between symptom duration subgroups. Thus, the observed statistical comparison results could differ in a sufficiently powered study. Investigating the effects of treatment in patients with early axSpA using an approach similar to that taken in rheumatoid arthritis clinical trials is warranted [27, 28]. 

The use of relatively short-term efficacy outcomes in this study does not exclude the possibility of differences emerging in the longer term, such as in structural damage progression. Due to limited research, the long-term evolution of efficacy outcomes across patients treated earlier versus later in their disease remains unclear, which could be addressed by future studies extending beyond 3 years. Additionally, analyses utilising a shorter symptom duration threshold (1 year) were of interest, as differences may be more pronounced in patients with a ≤ 1-year versus > 1-year symptom duration. However, these analyses were not feasible due to few patients having ≤ 1-year symptom duration. Further research utilising this threshold may shed light on an earlier window of opportunity in axSpA treatment and the effect of earlier treatment on disease outcomes, similar to findings in rheumatoid arthritis [9, 27]. 

In conclusion, this study demonstrates that regardless of symptom duration, bimekizumab treatment led to sustained improvements in disease activity, physical function, fatigue and HRQoL and sustained suppression of objective signs of inflammation to 2 years across the full disease spectrum of axSpA. Earlier versus later treatment of axSpA did not lead to different bimekizumab treatment effect at Week 16 after accounting for placebo. In contrast, significantly larger improvements in some 104-week outcomes were observed in the ≤ 5-year versus > 5-year symptom duration subgroup, although this cannot be definitively attributed to bimekizumab without a placebo comparator. These results highlight bimekizumab’s therapeutic potential for both early and more established axSpA. Future research using a shorter symptom duration threshold in larger populations may provide further insight into the effect of early bimekizumab treatment and whether a window of opportunity exists for the treatment of patients with axSpA. 

## Supplementary Information

Supplementary Material 1.

## Data Availability

Data are available on reasonable request. Underlying data from this manuscript may be requested by qualified researchers 6 months after product approval in the USA and/or Europe, or global development is discontinued, and 18 months after study completion. Investigators may request access to anonymised individual patient-level data and redacted trial documents which may include analysis-ready datasets, trial protocols, annotated case report forms, statistical analysis plans, dataset specifications and clinical study reports. Prior to the use of the data, proposals need to be approved by an independent review panel at www.Vivli.org and a signed data sharing agreement will need to be executed. All documents are available in English only, for a prespecified time, typically 12 months, on a password-protected portal.

## Abbreviations

ANCOVA: analysis of covariance
ASAS: Assessment of SpondyloArthritis international Society
ASAS40: Assessment of SpondyloArthritis international Society 40% response
ASDAS: Axial Spondyloarthritis Disease Activity Score
ASDAS-CII: ASDAS clinically important improvement
ASDAS-MI: ASDAS major improvement
ASQoL: Ankylosing Spondylitis Quality of Life
ASspiMRI-a: Ankylosing Spondylitis spinal Magnetic Resonance Imaging activity score
axSpA: axial spondyloarthritis
BASDAI: Bath Ankylosing Spondylitis Disease Activity Index
BASFI: Bath Ankylosing Spondylitis Functional Index
bDMARD: biologic synthetic disease-modifying antirheumatic drug
BL: baseline
BKZ: bimekizumab
BMI: body mass index
CfB: change from baseline
CI: confidence interval
CRP: C-reactive protein
csDMARD: conventional synthetic disease-modifying antirheumatic drug
DoS: duration of symptom
EMM: extra-musculoskeletal manifestation
HLA-B27: human leukocyte antigen B27
HRQoL: health-related quality of life
hs-CRP: high-sensitivity CRP
IBD: inflammatory bowel disease
IL: interleukin
LSM: least-square mean
MI: multiple imputation
mNY: modified New York
MRI: magnetic resonance imaging
nr-axSpA: non-radiographic axSpA
NRI: non-responder imputation
NSAID: non-steroidal anti-inflammatory drug
OC: observed case
OLE: open-label extension
OR: odds ratio
PBO: placebo
Q: question
Q4W: every 4 weeks
r-axSpA: radiographic axSpA
RD: relative difference
rOR: relative odds ratio
SD: standard deviation
SIJ: sacroiliac joint
SPARCC: Spondyloarthritis Research Consortium of Canada
TNF: tumour necrosis factor
TNFi: tumour necrosis factor inhibitor
TNFi-IR: prior inadequate response or intolerance to TNFIs
tsDMARD: targeted synthetic disease-modifying antirheumatic drug
yr: year
