# Supplementary material for: Impact of symptom duration on the short- and long-term efficacy of bimekizumab in axial spondyloarthritis: results up to 2 years
Source: Arthritis Res Ther. 2026 Jan 19;28:40. doi: 10.1186/s13075-026-03729-6 (PMC12896327; doi:10.1186/s13075-026-03729-6)
Supplement: Supplementary file 1 — Supplementary Material 1. [file 13075_2026_3729_MOESM1_ESM.docx]

**Impact of Symptom Duration on the Short- and Long-Term Efficacy of Bimekizumab in Axial Spondyloarthritis: Results up to 2 Years**

Sofia Ramiro,^1,2^ Fabian Proft,^3^ Raj Sengupta,^4^ Astrid van Tubergen,^5^ Anna Moltó,^6,7,8^ Lianne S. Gensler,^9^ Mitsumasa Kishimoto,^10^ Vanessa Taieb,^11^ Sarah Kavanagh,^12^ Shawna Evans,^12^ Victoria Navarro-Compán^13^

1. *Leiden University Medical Center, Department of Rheumatology, Leiden, The Netherlands*
2. *Zuyderland Medical Center, Heerlen, The Netherlands*
3. *Charité – Universitätsmedizin Berlin, corporate member of Freie Universität Berlin and Humboldt-Universität zu Berlin, Department of Gastroenterology, Infectiology and Rheumatology (including Nutrition Medicine), Berlin, Germany*
4. *The Royal National Hospital for Rheumatic Diseases, Bath, UK*
5. *Maastricht University Medical Center, Department of Medicine, Division of Rheumatology, Maastricht, The Netherlands*
6. *Université Paris Cité, Paris, France*
7. *Hôpital Bichat GHU AP-HP.Nord, Paris, France*
8. *INSERM U-1153 Center for Research in Epidemiology and Statistics, Paris, France*
9. *University of California, San Francisco, Department of Medicine/Rheumatology, San Francisco, California, USA*
10. *Department of Nephrology and Rheumatology, Kyorin University School of Medicine, Tokyo, Japan*
11. *UCB, Colombes, France*
12. *UCB, Morrisville, North Carolina, USA*
13. *La Paz University Hospital, IdiPaz, Department of Rheumatology, Madrid, Spain*

**Correspondence to:** Sofia Ramiro (Leiden University Medical Center, Department of Rheumatology, Leiden, The Netherlands and Zuyderland Medical Center, Heerlen, The Netherlands; sofiaramiro@gmail.com)

**Short title:** 2-Yr Efficacy by Symptom Duration Manuscript

**Trial registration:** NCT03928704 (BE MOBILE 1), NCT03928743 (BE MOBILE 2), NCT04436640 (BE MOVING)

**Funding:** UCB

**Key words:** axial spondyloarthritis, radiographic axial spondyloarthritis, IL-17 inhibitors, bimekizumab, symptom duration

SUPPLEMENTARY FIGURES

Supplementary Figure S1. Study design for BE MOBILE 1, BE MOBILE 2 and their combined open-label extension
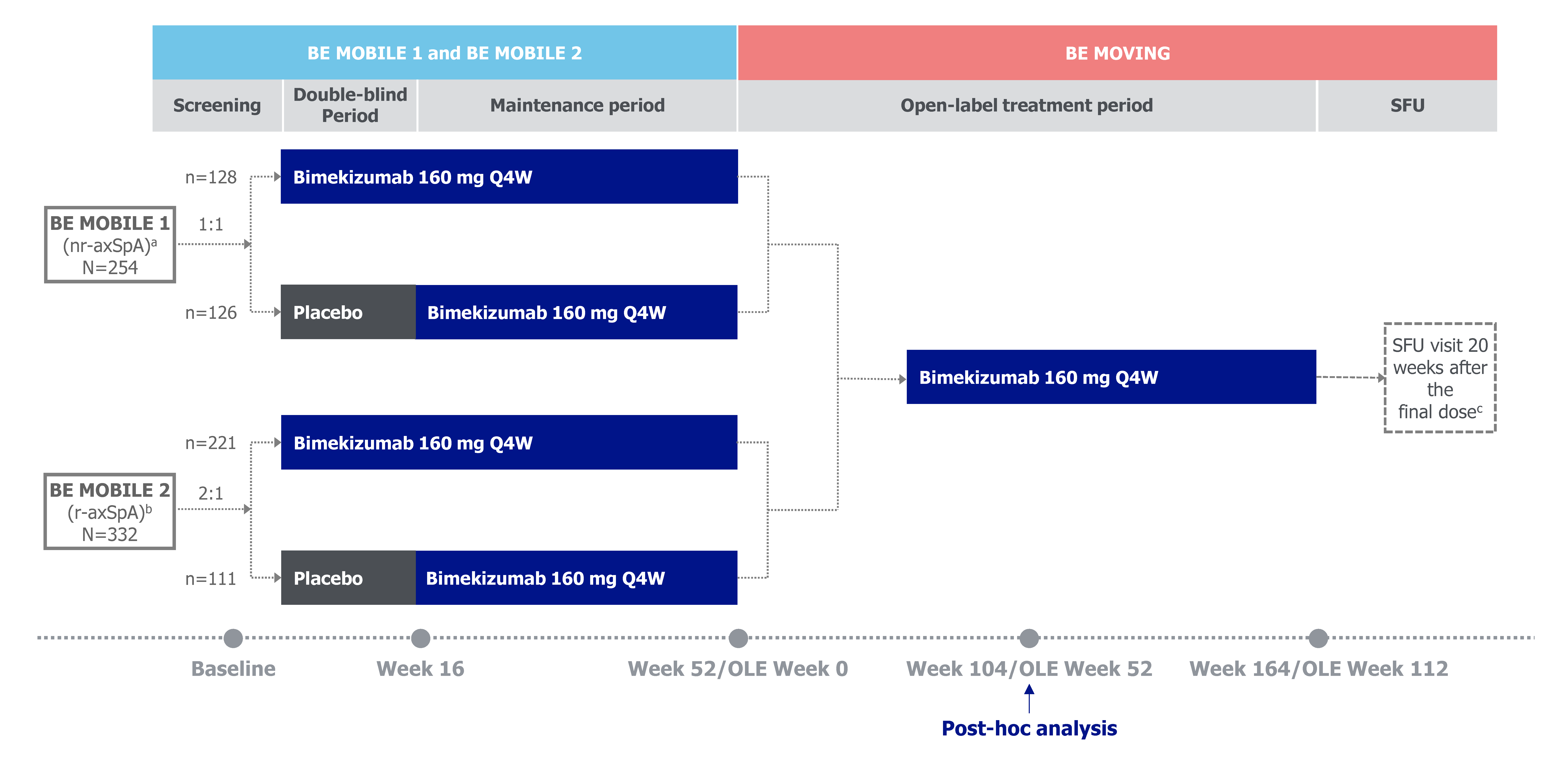


^a^Included patients with adult-onset nr-axSpA fulfilling ASAS classification criteria and objective signs of inflammation (active sacroiliitis on MRI and/or elevated CRP [≥6 mg/L]); ^b^Included patients with radiographic evidence of r-axSpA fulfilling modified New York criteria; ^c^Study participants will receive their final treatment dose at Week 160 (i.e., OLE Week 108); the SFU visit will be conducted 20 weeks after the final treatment. ASAS: Assessment of SpondyloArthritis international Society; axSpA: axial spondyloarthritis; CRP: C-reactive protein; MRI: magnetic resonance imaging; nr-axSpA: non-radiographic axial spondyloarthritis; OLE: open-label extension; Q4W: every 4 weeks; r-axSpA: radiographic axial spondyloarthritis; SFU: safety follow-up.

Supplementary Figure S2. Patient disposition and discontinuation reasons to Week 104


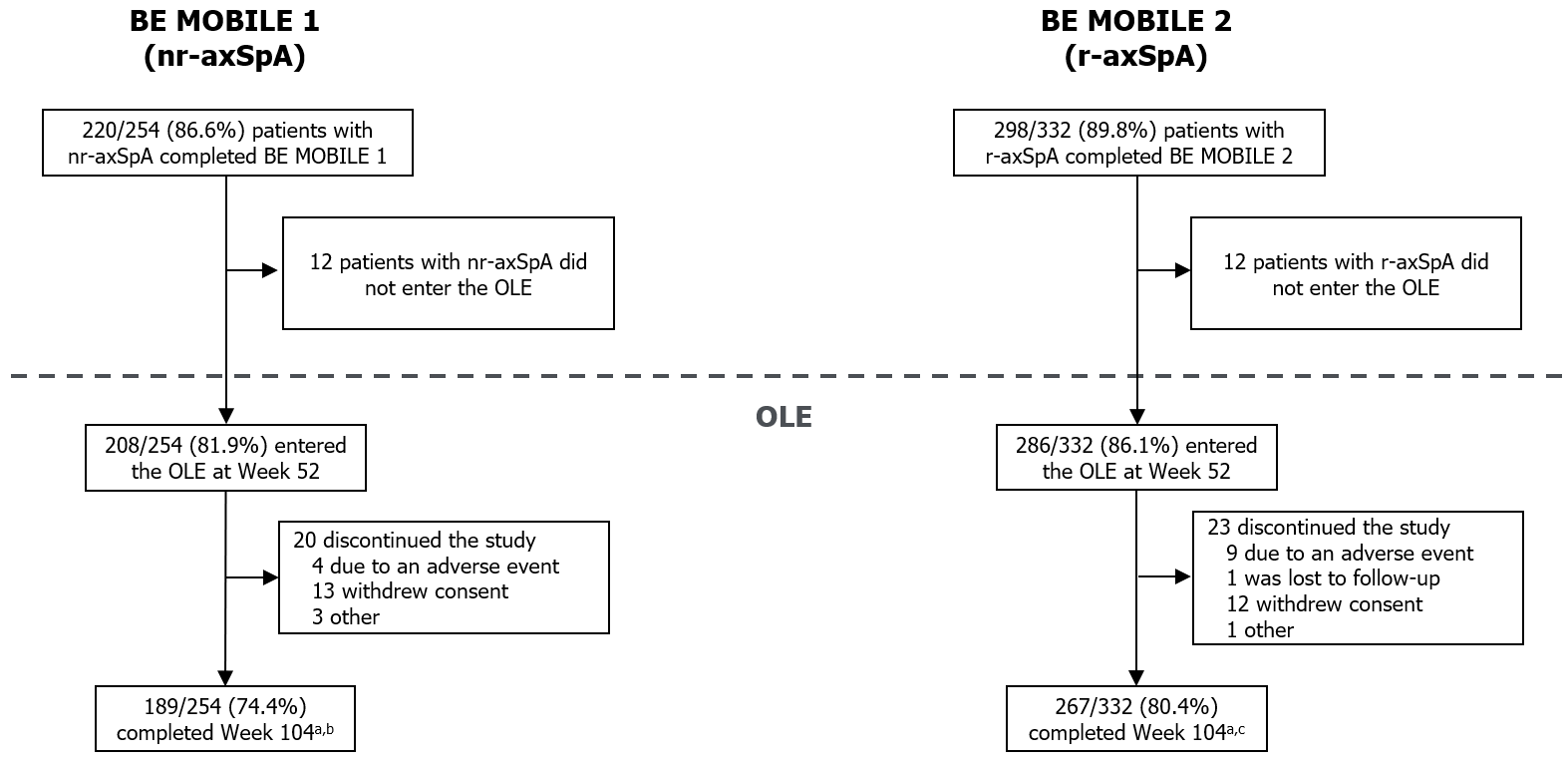


Randomised sets. Patient discontinuation reported as per the interim data cut in July 2023. ^a^Patients may have completed Week 104 but discontinued before the interim data cut. ^b^188 patients originally randomised to BE MOBILE 1 were still active in the study at the time of the interim data cut on 06 July 2023; ^c^263 patients originally randomised in BE MOBILE 2 were still active in the study at the time of the interim data cut on 06 July 2023. nr-axSpA: non-radiographic axial spondyloarthritis; OLE: open-label extension; r-axSpA: radiographic axial spondyloarthritis.

Supplementary Figure S3. ASDAS <1.3, ASDAS-CII, ASDAS-MI, disease activity (BASDAI) and fatigue (BASDAI Q1) stratified by symptom duration ≤5 and >5 years in BE MOBILE 1 and 2 (MI)








Randomised sets. N refers to the number of patients in their respective subgroups at baseline. Beyond Week 52, all data are pooled across bimekizumab- and placebo-randomised patients. ASDAS: Axial Spondyloarthritis Disease Activity Score; ASDAS-CII: ASDAS clinically important improvement; ASDAS-MI: ASDAS major improvement; axSpA: axial spondyloarthritis; BASDAI: Bath Ankylosing Spondylitis Disease Activity Index; BKZ: bimekizumab; CfB: change from baseline; DoS: duration of symptoms; MI: multiple imputation; nr-axSpA: non-radiographic axial spondyloarthritis; PBO: placebo; Q1: question one; r-axSpA: radiographic axial spondyloarthritis.
